# E-learning for self-management support: introducing blended learning for graduate students – a cohort study

**DOI:** 10.1186/s12909-018-1328-6

**Published:** 2018-09-24

**Authors:** Virginia Munro, Andrea Morello, Candice Oster, Christine Redmond, Anna Vnuk, Sheila Lennon, Sharon Lawn

**Affiliations:** 1 College of Medicine and Public Health, Flinders University, PO Box 2100, Adelaide, South Australia 5001 Australia; 2 Flinders Human Behaviour & Health Research Unit, Department of Psychiatry, Flinders University, PO Box 2100, Adelaide, South Australia 5001 Australia; 3 Discipline of Physiotherapy, School of Nursing & Health Sciences, Flinders University, PO Box 2100, Adelaide, South Australia 5001 Australia

**Keywords:** Self-management, Self-management support, E-learning, Blended learning, Virtual learning, Chronic care, Person-centred care

## Abstract

**Background:**

E-learning allows delivery of education in many diverse settings and researchers have demonstrated it can be as effective as learning conducted in traditional face-to-face settings. However, there are particular practices and skills needed in the area of providing patient self-management support (SMS), that may not be achievable online. The aim of this study was to compare three approaches in the training of university students regarding the preparation of a Chronic Condition Self-Management Care Plan: 1) traditional face-to-face delivery of SMS training, 2) an e-learning approach and 3) a blended approach (combining e-learning and face-to-face teaching).

**Methods:**

Graduate entry physiotherapy students and medical students at Flinders University were recruited. Depending on the cohort, students were either exposed to traditional face-to-face training, e-learning or a blended model. Outcomes were compared between the three groups. We measured adherence to care plan processes in the preparation of an assessment piece using the Flinders Program Chronic Care Self Management tools. A total of 183 care plans were included (102 traditional, 52 blended, 29 e-learning,). All students submitted the Flinders Program Chronic Care Plan for university assessment and these were later assessed for quality by researchers. The submission was also assigned a consumer engagement score and a global competence score as these are integral to successful delivery of SMS and represent the patient perspective.

**Results:**

The blended group performed significantly better than the traditional group in quality use of the Flinders Program tools: Problem and Goals (*P* < 0.0001). They also performed significantly better in the total care plan score (*P* < 0.0001) and engagement score (*P* < 0.0001). There was no significant difference between the groups for the Partners in Health tool.

**Conclusions:**

In this pilot study, the blended learning model was a more effective method for teaching self-management skills than the traditional group, as assessed in the development of a chronic condition self-management care plan. We anticipate that future research with identical groups of students would yield similar results but in the meantime, academics can have confidence that blended learning is at least as effective as traditional learning methods.

## Background

Education for medical and allied health students has undergone significant changes. In particular, advances have been made in both technologies available for education and in the ability of students to navigate new technology. The utilisation of such technology can overcome barriers to learning such as geographical isolation and limited specialist training staff, whilst providing a standardization of experience [1]. One of the technologies is e-learning. E-learning is the delivery of education through Information and Communication Technology (ITC) using a wide variety of instructional designs and formats, and includes both synchronous (real time, e.g., videoconferencing) and asynchronous (self-paced) learning. Many things can constitute e-learning, including CD-ROMs, discussion boards, virtual patients and webinars. Central to the definition is the use of the Internet and computer to deliver information and interact with the learner, to partly replace the human instructor. Importantly, e-learning does not rely on a human instructor being present at the time that the student is learning [2].

There has been much research around the implementation of physical simulations for medical and allied health students with the emergence of digital simulation technologies enabling learners to see, interact and learn in representations of scenarios that were previously impossible [3]. There is a growing body of evidence to suggest that clinical skills acquired in medical simulation laboratory settings transfer directly to improved patient care practices and better patient outcomes [4, 5]. However, there are limited studies on the effectiveness of e-learning outside the area of clinical and procedural skills acquisition, for example, chronic condition self-management support and other contexts that aim to develop the learner’s person-centered care skills [6–9]. The use of e-learning in such areas may be challenging.

Chronic disease affects many people throughout their lives, and yet they spend less than 1% of their life within the healthcare system with the rest spent living with the realities of chronic disease on a daily basis [10]. Educating and supporting patients’ mastery of self-management skills can lead to significant improvement in health [11]. In self-management, patients are encouraged to partner with health professionals and other supports to assess their healthcare needs and carry out the needed tasks for managing their chronic conditions. Self-management has been defined as ‘the individual’s ability to manage the symptoms, treatment, physical and psychosocial consequences and lifestyle changes inherent in living with a chronic condition’ [11]. It requires patients to have a specific set of skills around ongoing assessment and awareness, goal setting and problem solving [12, 13]. However, it may be difficult for patients to achieve alone and they may benefit from self-management support (SMS) provided by others (e.g., health professionals, family, carers). SMS includes the provision of education and information to improve health literacy, practical support, goal setting and achievement but also includes holistic support from service providers, family and friends.

The provision of SMS by health professionals requires effective training and education, particularly the development of effective communication and person-centered skills to facilitate engagement and behaviour change. Lawn et al. (2017) argue that training provided to these health professionals is best conducted with formats that maximise the uptake of these skills and suggest mediums involving significant experiential learning, practice, critical reflection and feedback would be appropriate [14]. They suggest that while evidence for effectiveness of e-learning is growing, it is unclear which e-learning instructional designs and formats are best for teaching the skills necessary for working with patients with chronic care needs.

Lawn et al., (2017) conducted an integrative review of e-learning in the delivery of SMS training for health professionals. Overall, they found that few of the e-learning approaches reviewed provide the high levels of interactivity, reflection, practice and application required for health professionals to learn SMS [14]. Given that chronic care requires the development of partnerships and person-centred care, they suggest that improved results could be achieved by using a blended model that combines e-learning and face-to-face teaching.

To date, only one study exists that has demonstrated web-based SMS training for health professionals which was both feasible and able to positively support patient self- management. Yank et al., (2013) provided self-management training via four Webinars to 57 primary care residents and providers across the United States. This webinar was given on a weekly basis. Between sessions participants applied the learned techniques to clinical patients. Both participants and residents showed positive results regarding the webinar in their attitude: the residents demonstrated positive changes on all belief questions (P 0.001–0.012), and all participants demonstrated significant increases on confidence questions regarding their ability to support self-management (*P* < 0.01). However, this study did not look at behavioural changes.

To help address this knowledge gap, the current pilot study aimed to compare traditional, e-learning and blended self-management training programs for future health professionals.

## Methods

### Aim

The aim of this study was to compare traditional face-to-face delivery of SMS training with both an e-learning approach and a blended approach in the training of university students regarding the preparation of a Chronic Condition Self-Management Care Plan.

### Participants

Participants were students in the Flinders University Graduate Entry Medical Program (GEMP) in Adelaide and Darwin (3000 kms from Adelaide) and the Master of Physiotherapy program (MPT) in Adelaide. The participants were all students who had not previously received formal SMS training in their postgraduate courses. Both programs are graduate entry with students coming from a wide variety of backgrounds. All students were in the second year of study, with the GEMP and MPT programs being 4 and 2 years long, respectively and all students studying these courses were included. All groups had completed a clinical placement but the MPT in the blended group were in their final year of study.

### Intervention

As part of their studies, all students were required to undertake training in the Flinders Chronic Condition Management Program [15–17].

This program teaches a structured assessment approach, using processes and tools that explore self-management skills in partnership with patients, leading to the formulation of a self-management care plan (Table 1).

**Table 1:** Steps of the Flinders Program

Partners in Health Scale	A self-reported 12 item questionnaire which assesses seven principles of attributes of a person’s self-management [26]. It is a patient assessed Likert-rated validated questionnaire informed by the WHO and Australian National Chronic Disease Strategy principles of self-management. It enables measurement of perceived change over time where 0 = less favourable and 8 = more favourable self-management capacity.
Cue and Response Interview	An adjunct to the PIH using open-ended questions or cues for the health worker to explore the patient’s responses to the PIH in more depth, with the patient and worker comparing their Likert-ratings to identify agreed good self-management, agreed issues to be addressed, and any discrepancies in views that can then be discussed as part of formulation of a self-management care plan. It enables the strengths and barriers to self-management to be explored from perspectives of both the patient and the health worker, and checks assumptions that either party may have, as part of a motivational process.
Problem and Goals Assessment	Defines a problem statement from the patient’s perspective (the problem, its impact and how it makes them feel) and identifies action-based specific, measurable, achievable, realistic and timely (SMART) behavioural goals that the patient can work towards. It is Likert-rated, allowing measurement of progress over time where 0 = not a problem and 8 = a significant problem; and goal statements: 0 = no progress towards achievement and 8 = achieved.
Chronic Condition Management Care Plan	Includes self-management issues, aims, steps to achieve them, who is responsible and date for review.

All GEMP students based in Adelaide received the traditional face-to-face delivery method, those based in Darwin received the e-learning delivery and the MPT students received a blended program (Table 2). The material was presented to the students either face-to-face by an academic in a traditional lecture theatre format of 3 h duration or via access to an online module. This online module was either accessed as a group with a supervisor present by the e-learning group, or independently in the blended group. All groups then role-played different scenarios; the GEMP students conducted the role plays in student pairs while the blended MPT group role played with volunteer patients who were able to provide ad-hoc informal feedback, from their experience as patients.

**Table 2:** Flowchart of student learning in various study groups (GEMP = graduate entry medical program, MPT = Master of Physiotherapy)

Group	GEMP Adelaide (*n* = 102)	GEMP Darwin (*n* = 29)	MPT Adelaide (*n* = 52)
Intervention	Traditional learning model (face to face)	e-learning model	Blended learning model
Module delivery	Module delivered by academic in lecture style format (3 h)	Online module with coordinator present in room	Online module accessed independently
Practice	Role playing in pairs	Role playing in pairs	Role playing with volunteer patients, with feedback
Feedback	Lecture with academic (3 h)	Videoconference with academic (3 h)	Lecture with academic (3 h)

After delivery of the content and completion of a role-playing session, a feedback session was conducted either in a 3 h lecture style format with the academic being present for the traditional and blended groups, or via videoconference with the academic for the e-learning group. This feedback session provided students with the opportunity to reconcile their knowledge of providing SMS. It was an opportunity to address any questions from the students and ensure that the students understood the assessment task. The volunteer patients were not present for this feedback session and the same academic performed the feedback session for all three groups.

For their assessment, students utilized the Flinders model together with a patient.

Students then had to submit the set of Flinders tools used in care planning along with a completed care plan for assessment. For the GEMP students, the care plan was assessed on a non-graded pass or fail basis, whereas for the MPT students it was a graded assessment that included a self-reflection on the Flinders Program process.

### Data collection and scoring

Scoring the patient-centered care plan assessed students’ competency in adherence to effective care plan processes. The care plans were graded according to a rubric (Table 3). The grid was determined with external expert input by a team with considerable experience in care plan processes. It was drawn from a framework routinely used to determine competence for students and health professionals undertaking Flinders Program training in Australia and overseas.

**Table 3:** Scoring grid used for individual care plans (Note: SMART – Specific, measurable, achievable, realistic and timely)

Partner in Health					
No. of questions answered	0 (no questions)	1 (1–11)	2 (all questions)		
Cue and Response					
Scored by health practitioner	0 (none)	1 (some)	2 (all)		
Scored by client	0 (none)	1 (some)	2 (all)		
Notes provided around scores < 4 or discrepancy > 3	0 (none)	1 (some)	2 (all)		
Enough notes on discussion of scores	0 (no notes)	1	2 (limited notes)	3	4 (detailed notes)
Notes are written from client’s perspective	0 (none)	1	2 (reflects client perspective)	3	4 (client’s own words)
Identified issues transferred to care plan	0 (none)	1 (some)	2 (all)		
Problem and Goals					
Problem statement	0 (neither)	1	2 (succinct or first person)	3	4 (succinct and first person)
Response to problem	0 (none)	1	2 (reflects client perspective)	3	4 (client’s own words)
Appropriate impact response	0 (absent)	1	2	3	4 (describes what happens)
Appropriate feeling response	0 (absent)	1	2	3	4 (describes feelings)
Problem statement	0 (no response)	1	2	3	4 (all response to open questions are reflected)
Client scored problem	0 (none)	1 (scored 0,1,2)	2 (scores > 3)		
Medium to long-term goal statement	0 (no response)	1	2	3	4 (linked to problem statement
Goal	0 (absent)	1 (either positive or regular activity)	2 (both positive and regular)		
SMART goal	0 (absent)	1 (some elements)	2 (all elements)		
Client scored goal	0 (no score)	1 (scored 6, 7 or 8)	2 (scored < 6)		
Care Plan					
Transfer of problem statement and score	0 (absent)	1 (transferred problem statement or score)	2 (transferred both elements)		
Transfer of goal statement and score	0 (absent)	1 (transferred goal statement or score)	2 (transferred both elements)		
Language	0 (technical language and jargon)	1	2	3	4 (client’s own words, avoiding technical language and jargon)
Management aims – what client wants to achieve	0 (none)	1	2 (not clinically focused)	3	4 (present)
Manageable Interventions	0 (none)	1	2	3	4 (small steps)
Client’s name	0 (absent)	1	2 (present)		
Review date	0 (absent)	1 (some)	2 (all)		

All care plans were de-identified and assigned a coding to distinguish the groups. All researchers were blinded to the group of the care plan. A single researcher trained in the correct development of the care plan but not involved in the delivery of the course scored the care plans. Scores were assigned to submitted care plans in each group, which often represented 2 students working in pairs. Scores were assigned for each section (see Table 3); the maximum score for the Partners in Health scale was 2, for the Cue and Response Interview was 16, the Problems and Goals was 34 and Care Plan was 20. Total score was a maximum of 72 points.

Two additional researchers independently graded the care plans for other important features that demonstrated effective care plan processes. One of these researchers (an accredited trainer in the use of the Flinders Program tools and consumer advocate) assigned an engagement score. This engagement score (out of 8) reflected the consumer perspective with 1 representing poor perceived engagement with the patient and 8 representing excellent perceived engagement. The other researcher (also accredited trainer in the use of the Flinders Program tools) assigned a global competence score that reflected the health professional perspective and perceived overall competence of the student (out of 8).

### Correlation between outcome measures

We examined the correlation between the scores obtained from the Flinders Care Plan, the engagement score and the global score across the three delivery methods. This was done to determine if there was internal consistency between the three different methods of grading the students’ Care Plans. The correlation matrix shows a highly significant relationship (*r* = 0.38–0.43, *P* < 0.0001) between all three scores (Table 4) indicating internal validity in scores assigned for each measure. The rho suggests a weak-moderate correlation, which is expected as each score assesses different attributes of the care planning process.

**Table 4:** Differences in adherence to care plan processes by educational delivery method (*denotes statistically significant difference *P* < 0.01, #post-hoc test not computed because overall between-subject effect was > 0.001)

Element Assessed (overall *P* value between groups)	Scores Mean (SD)			*P* value from LSD post-hoc test		
	A Face-to-face (*n* = 102)	B Blended (*n* = 52)	C e-learning (*n* = 29)	A vs B	A vs C	B vs C
Partners in Health (*P* = 0.2)	1.9 (0.19)	2 (0)	2 (0)	Not computed^#^		
Cue and Response (*P* = 0.002)	10.9 (3.3)	12.7 (2.3)	12.4 (4)	Not computed^#^		
Problems and Goals (*P* = < 0.0001)	23.9 (7.2)	28.8 (7)	27 (6)	< 0.0001*	0.037	0.248
Care Plan (*P* = 0.001)	13.6 (5.4)	16.6 (3.9)	15.5 (3.6)	< 0.0001*	0.06	0.303
Total score (*P* = 0.001)	50.3 (11.7)	60.1 (9.8)	56.9 (10)	< 0.0001*	0.012	0.746
Engagement score (*P* = < 0.0001)	5.1 (1.1)	6.1 (1.1)	5.8 (0.94)	< 0.0001*	0.005*	0.204
Global score (*P* = 0.01)	4.6 (1.2)	5.2 (1)	4.7 (1.4)	Not computed^#^		

### Data analysis

Statistical analyses were performed using SPSS Statistics version 19 (IBM, Armonk, USA). Since we found that the data displayed approximate normal distributions, it was tenable to use parametric tests (multivariate analysis of variance with the LSD post-hoc test if overall between-subject effects was < 0.001 and Pearson Correlation coefficients). A type 1 error rate of alpha = 0.01 was used in all analyses to test for statistical significance due to multiple testing with the LSD test and reduce the likelihood of type 1 error.

## Results

### Differences between groups for outcome measures

Between-group analyses were conducted for each of the care plan domains (Table 5). The group that received a blended model of training (group B) had a significantly higher score for Problem and Goals (*P* < 0.0001), Care Plan (*P* < 0.0001), Total score (*P* < 0.0001), and engagement score (*P* < 0.0001) compared to the traditional face-to-face group (group A). There were no significant differences between the blended (group B) and the e-learning group (group C) across any of the domains. There was a significant difference for engagement score between the traditional face-to-face (group A) and e-learning group (group C) (*P* = 0.005), with the e-learning group (group C) scoring significantly higher; the other domains were not significantly different.

**Table 5:** Correlation betweeCorrelation between total care plan score, engagement score and global score across all groups

		Engagement Score	Global Score
Total Care Plan Score	Pearson correlation	0.386	0.429
	Sig. (2-tailed)	< 0.0001^a^	< 0.0001^a^
	*n*	183	183
Engagement Score	Pearson correlation		0.388
	Sig. (2-tailed)		< 0.0001^a^
	*n*		183

^a^Statistically significant finding at the level of *P* < 0.01

## Discussion

We report on the first study to compare the effect of traditional face-to-face, e-learning and blended learning in Self-Management Support (SMS) training. SMS requires particular skills surrounding patient-centered care and communication. It was unclear whether the use of online modules would facilitate the development of these particular skills sets, which are traditionally taught face-to-face. Our results, however, have demonstrated that the e-learning module can enable participants’ learning with the advantage that educational programs are not constrained by the need to provide face-to-face teaching for the module delivery.

The findings showed a magnitude of difference between groups in favour of the blended learning group. Blended learning combines online learning components with face-to-face learning and practice. While the experience of developing e-learning was relatively new in the teaching unit, the study results revealed that the program was feasible and enhanced effectiveness.

SMS requires a particular set of skills surrounding communication and being patient centered [18] and there is limited evidence for the effectiveness of e-learning when teaching person-centered skills. A recent integrative review found that it was unclear whether online modules would be useful in this field [14]. However the results of this study are supported by the findings of a recent large meta-analysis by the United States Department of Education, which concluded that blended learning was significantly more effective than face-to-face or online courses [19]. This review was of education generally, not specifically in the area of SMS but it has applicability. The authors caution that the results do not suggest that online learning is superior as a medium but that the advantages of blended learning that leads to superior learning are due to a combination of elements in the teaching conditions (which likely include additional learning time and materials, as well as opportunities for collaboration).

Constructivist learning principles highlight how students create their own meaning of knowledge [20] and this is enabled by blended learning where students are more able to self regulate and to have more control of their learning [21]. One of the reasons blended courses are significantly more effective than completely face-to-face or online courses in education generally, is that students feel more prepared for classroom activities due to the greater opportunity to reflect and review the material online [22]. Welch et al. stressed that it was important to include opportunities to practice face-to-face following online training for motivational interviewing [23]. They found that when nurses completed a web-based learning module in preparation for context-based skills training, there was a significant increase in knowledge scores, combined with strong positive attitudes prior to and following the module.

A recent review by Lawn et al., 2017 found that while e-learning has many benefits, complete online delivery of SMS poses some problems with regards to the need to work with patients in training. This review identified the need for practice and application opportunities, plus time for reflection to be built into the learning process. Other studies have found that some chronic conditions require face-to-face delivery to demonstrate and practice SMS skills [6]. The use of a blended approach in this study allowed for learners to access material and process material in their own time, which was then followed up with direct person training. This follows the Knowledge- Process-Practice model proposed by Shaw et al., 2015 to ensure behaviourist and cognitivist requirements of education are met within the content of e-learning materials [24].

The rurally located GEMP students received their teaching via e-learning. This group was small (*n* = 29); therefore it is difficult to make extrapolations, however they had a significantly higher engagement score (*P* = 0.005) compared to the traditional group. Similarly, while not significant at the *P* = 0.01 level, they scored better in each of the other domains compared to the face-to-face control group. This was not an anticipated result because previous researchers have suggested that e-learning might be a less appropriate teaching medium for learners to acquire complex emotional skills such as those required in SMS. However, active engagement and participation seems to be the key factor for successful learning [24]. It is possible that these students in a rural context, although more isolated, had a more supportive network with particular implications for the development of the care plans.

The majority of studies evaluating the use of the Internet for medical education have also included evaluation on the satisfaction of participants [25]. While this is an important concern as the Internet grows as a medium for education, it is initially necessary to determine if such systems are effective ways to enhance practice. In the current study, we did not measure the students’ satisfaction but examined if implementation of e-learning could be used in the training of students in SMS for successful skill development for improved patient care, with subsequent studies required to determine participant satisfaction.

In the current study, assessment was graded using several scores measuring different aspects of the Flinders Program. These scores looked at adherence to care plan processes for each of the Flinders Program tools and also scores of consumer engagement and global competency. The results for these scores were correlated, showing agreement between the quality of the submitted work and ratings for being patient-centred and demonstrating active engagement with the material. In our study, comparing traditional face-to-face, blended and e-learning in the delivery of content and skills for providing SMS to patients, the students in the blended group demonstrated the greatest ability to provide SMS in chronic care self-management across all scores: care plan development, engagement and global score. Future studies are required, with more identical groups, to determine which aspects of SMS are best taught online and to determine the student opinion on such a teaching format.

### Study limitations

Our study has several potential limitations. Firstly, the three groups were not identical in clinical or educational experience. While all students were post-graduate health professional trainees in the second year of their post-graduate qualification, they differed in that the MPT students were in the final year of their qualification and hence were already working at a junior health professional level in their clinical placements while the medical students had only just started clinical placements, with two more years of their degree to be done. Additionally, as postgraduate students, their previous work, life and study experiences were varied and diverse and we did not formally assess the baseline level of participants’ exposure to SMS training to determine if this was significant or not. Secondly, the expectations of assessment tasks were not identical for each group; although the assignment was compulsory for all groups, the MPT group’s assessment was formally graded and inclusive of a self-reflective component, while the other two groups received a non-graded pass, which may have influenced the effort given to the assessment. However, while the care plan was assessed on a pass/fail basis for the medical students, the end of year practical examination (OSCE) included one station based on the Flinders Program, therefore there was still an assessment-based incentive for all students surrounding the learning of SMS, but not the Care Plan assessment piece used for this study. Thirdly, the scoring itself was not externally validated by another assessor but was completed by a single investigator blinded to the cohort; however, two independent trainers provided a global competence score for each care plan (one trainer provided the consumer score and one provided the engagement score) and these scores all correlated. Fourthly, a major limitation with this study is that the blended group had access to the online study modules, therefore were potentially able to spend more time learning the subject. Fifthly, the blended students practised with volunteer patients while the other students practised on each other, which may have resulted in some learning differences. However, all students were required to undertake complement of the Care Plan with an actual patient. Finally, this study did not assess the patient’s perspective of engagement, which is an essential part of the Flinders Program. While a proxy score was assigned by one of the assessors, a score from the actual patients’ interview may have yielded different results.

## Conclusions

This pilot study, utilized two different groups of students, to determine if a blended teaching format could be used to implement the delivery of SMS for the skills taught in the Flinders Program of chronic care. The findings suggest that a blended teaching format can be utilized with the confidence that it is at least as effective as the traditional face-to-face medium.

There are a number of recognised resource-based advantages for e-learning such as time and travel costs and classroom-learner-staff availability. However, other advantages, such as the ability to be self-paced and traceable, catering to different learning styles and allowing the learner to review as needed are also of benefit to the learner. A teaching format that allows for reflection and then practice may be a more successful tool than traditional face-to-face teaching in SMS. The delivery of SMS education using a blended approach provided students with the time to reflect on the material as well as opportunities for active engagement and ultimately successful preparation of a care plan utilising the Flinders Program. Studies are also required to determine how the delivery of SMS training affects the patient perspective.

## Abbreviations

GEMP: Graduate entry medical program
ITC: Information and communication technology
MPT: Master of physiotherapy
PIH: Partners in health
SMART: Specific, measurable, achievable, realistic and timely
SMS: Self-management support
